# Evaluation of Family-Based Interventions as a Therapeutic Tool in the Modulation of Childhood Obesity: A Systematic Review

**DOI:** 10.3390/children11080930

**Published:** 2024-07-31

**Authors:** Diego Fernández-Lázaro, Ana M. Celorrio San Miguel, Evelina Garrosa, Ana M. Fernández-Araque, Juan Mielgo-Ayuso, Enrique Roche, Soledad Arribalzaga

**Affiliations:** 1 Department of Cellular Biology, Genetic, Histology and Pharmacology, Faculty of Health Sciences, University of Valladolid, Campus de Soria, 42003 Soria, Spain; 2 Neurobiology Research Group, Faculty of Medicine, University of Valladolid, 47005 Valladolid, Spain; 3 “Nutrition for Sport and Exercise” Working Group, Spanish Nutrition Society (SEÑ), 28010 Madrid, Spain; jfmielgo@ubu.es (J.M.-A.); eroche@umh.es (E.R.); 4 Doctoral School, University of León, Campus de Vegazana, 24071 León, Spain; amcelorrio@educa.jcyl.es; 5 Faculty of Psychology, University of Salamanca, 37007 Salamanca, Spain; evelinags17@gmail.com; 6 Department of Nursing, Faculty of Health Sciences, University of Valladolid, Campus de Soria, 42003 Soria, Spain; anamaria.fernandez@uva.es; 7 Pharmacogenetics, Cancer Genetics, Genetic Polymorphisms and Pharmacoepidemiology Research Group, Faculty of Health Sciences, University of Valladolid, Campus de Soria, 42003 Soria, Spain; 8 Department of Health Sciences, Faculty of Health Sciences, University of Burgos, 09001 Burgos, Spain; 9 Department of Applied Biology-Nutrition, Institute of Bioengineering, University Miguel Hernández, 03202 Elche, Spain; 10 Alicante Institute for Health and Biomedical Research (ISABIAL), 03010 Alicante, Spain; 11 CIBER Fisiopatología de la Obesidad y Nutrición (CIBEROBN), Instituto de Salud Carlos III (ISCIII), 28029 Madrid, Spain; 12 Faculty of Sport Sciences, European University of Madrid, Villaviciosa de Odón, 28670 Madrid, Spain; mariasoledad.arribalzaga@universidadeuropea.es

**Keywords:** anthropometric parameters, childhood obesity, family-based interventions, nutrition, physical parameters, systematic review

## Abstract

Childhood obesity is a major public health burden. The prevalence of weight excess for children and the adolescent population (8 to 16 years) is 34.9%. During childhood, lifestyles are acquired, which are developed in adulthood. In this context, the role of parents is crucial, since they are the model to imitate. We aimed to evaluate the current evidence on the effects of family-based interventions as a tool in the treatment of childhood obesity. We reviewed studies indexed in several databases according to the Preferred Reporting Items for Systematic Reviews and Meta-Analyses (PRISMA) guidelines. Original articles published from 1 January 2014 to 30 May 2024 with a controlled trial design were considered, in which family-based interventions were carried out compared to a control group or to data before the intervention. Although 148 records were identified in the search, 6 studies met inclusion criteria. Overall, studies reported beneficial effects of family-based interventions on improving anthropometric parameters: BMI z-score, BMI, waist circumference, and body fat percentage. Regarding nutritional and physical activity guidelines, general recommendations must consider increased consumption of fruits and vegetables, reducing sugary drinks, controlled screen time, and 30–60 min of physical activity/day. Thus, family-based interventions could be an effective non-pharmacological strategy for modulating childhood obesity, allowing families to modify their lifestyles.

## 1. Introduction

The significant global increase in obesity is one of the most difficult public health problems that today’s society must face. This situation not only affects countries with higher incomes but also is increasing in countries with low and middle incomes [1]. According to the World Health Organization (WHO), between 2010 and 2022, the worldwide prevalence of obesity (body mass index (BMI) > 30 kg/m^2^) nearly doubled [2]. Thus, the prevalence of weight excess for children and the adolescent population (8 to 16 years) is 34.9%, at 20.7% for overweight and 14.2% for obesity (BMI > 30 kg/m^2^). The increasing tendency has allowed researchers to estimate that in 2028, 2.7 billion adults and 268 million school-age children will be overweight or obese. In the world, overweight and obesity are associated with more deaths than underweight [3]. Obesity is a chronic disease that usually begins in childhood and adolescence. According to the WHO, childhood obesity is one of the most serious public health challenges worldwide in the 21st century and is progressively advancing, especially in the urban environment [4]. In fact, obesity is the most frequent nutritional disorder in children and adolescents. In the ALADINO study [5], the prevalence of overweight in boys presented a range of 14 to 26% and 13 to 25% in girls. The results are consistent with those of other studies that have already shown an increase in the prevalence of childhood obesity, such as the EnKid study [6], the National Health Survey in minors in Spain [7], and ENE-COVID [8].

Childhood overweight and obesity are due to multiple causes, in which genetic, hormonal factors, lifestyles, and environmental influences come together. However, lifestyles have the greatest influence. In this context, the preventive programs that are adopted to reduce overweight and obesity at an early age acquire central importance. The treatment of childhood obesity requires combining a non-deficient and balanced diet that allows adequate growth, increased physical activity, and the modification of eating attitudes and behaviors both of the child and the family environment [9,10,11]. Psychological help should be considered to treat specific aspects, such as low self-esteem, traits of anxiety and depression, or the harassment that obese children often suffer, making their social integration and emotional development in the short and long term difficult [12,13].

Today’s children are growing up in a culture that places a high value on physical appearance and views obesity as a sign of laziness, weakness, and selfishness [7]. Consequently, it is easy to overlook the emotional consequences faced by children and adolescents who are overweight or obese. Although obesity is not included in psychiatric disorders, it can manifest severe psychological symptoms, such as anxiety, feelings of worthlessness, low self-esteem, aggression, social segregation, depression, and even suicidal tendencies. Therefore, the rapid increase in obesity in children is coming together with mental health disorders [14]. In this context, interventions must be directed toward the psychological and nutritional state of children. Additionally, interventions should contribute to reducing social biases toward obesity, helping to develop effective mechanisms in obese children to manage stress by increasing positive relationships and emphasizing encouraging lifestyle changes [15].

Obesity should be treated as a chronic health problem to achieve a stable change that affects lifestyles and, at the same time, emotional states. The most difficult goal in managing obesity is not losing weight but maintaining the achieved reduction. The evidence shows that the most effective obesity treatment must be multidisciplinary and coordinated, based on diet, psychotherapy, and physical activity [16]. In this context, family-based interventions (FBIs) are a type of research-backed treatment for childhood obesity that can be focused on diet, physical activity, behavior modification, and parenting skills to support weight loss [16]. FBIs have been developed and refined over the past 35 years, consistently demonstrating reliable improvement in children’s weight outcomes [17]. Family-based behavioral treatment for weight control demonstrates effectiveness among overweight or obese school-age children 6–12 years old [17]. However, very few children receive this intensive treatment partly due to the high costs of administration [18].

The central aspect of FBIs is the traffic light system, utilized to categorize foods and activities. Foods and activities in red color are high-energy-dense foods and screen time, respectively, while foods and activities in green are low-energy-dense foods (fruits and vegetables) and moderate to vigorous physical activity [19]. Changing the home environment is one of the goals of FBIs. The reviews provided on the importance of the family in child obesity interventions are old, and the present report will update the latest approach [20]. Altogether, the purpose of this systematic review was to test the effects of FBIs as a tool in the treatment of childhood obesity. We hypothesized that FBIs could improve physical and/or anthropometric parameters in comparison to standard interventions carried out in randomized controlled clinical trials ignoring the family environment.

## 2. Materials and Methods

### 2.1. Search Strategy

This systematic review was carried out according to the Preferred Reporting Items for Systematic Review and Meta-Analyses (PRISMA^®^) guidelines [21]. The review was registered in the OSF repository (https://osf.io/zyadb (accessed on 4 June 2024)). The PICOS [22] model was used to define the criteria for inclusion: P (population): “children under 16 years of age who are overweight and/or obese”; I (intervention): “family-based interventions”; C (comparison): “same conditions with/without family-based intervention”; O (outcomes): “physical and/or anthropometric modifications”; and S (study): “randomized controlled clinical trials”.

A structured search of the Web of Science (WOS), Cochrane, Medline (Pubmed), and Scielo was of high quality and guaranteed suitable bibliographic support for this systematic review completed on 4 June 2024. The keywords provided in the full article were used following this Boolean search equation: (“childhood obesity” OR “obesity” OR “children”) AND (“physical activity” OR “exercise”) AND (“intervention” OR “family-based intervention”) (Appendix A).

Relevant articles were also obtained using this equation by applying the snowball strategy, with all titles and abstracts from the search being cross-referenced to identify duplicates and any potentially missing studies. Titles and abstracts were selected for further review of the full text. The search for published studies was independently performed by 2 authors (D.F.-L and S.A.), and disagreements about records were resolved by a third reviewer (A.M.C.S.M).

### 2.2. Inclusion Criteria

The following inclusion criteria were applied in selecting studies for the articles found in the search: (I) studies using family-based interventions on obesity/overweight in children; (II) studies including a similar control group in obese/overweight children with/without family-based interventions; (III) publications with human samples under 16 years of age; (IV) records written in German, French, Italian, Spanish, Portuguese, or English; (V) studies published in the period from 1 January 2014 to 30 May 2024; (VI) studies evaluating physical and/or anthropometric modifications as outcomes (primary or secondary); (VII) randomized controlled clinical trials; (VIII) records that have a score equal to or greater than 8 on the McMaster Critical Review Form; and (IX) original studies, excluding systematic reviews, narrative reviews, notes, theses, dissertations, reviews, conference abstracts and proceedings, and other non-original studies. The chosen time (last 10 years) presents a significant evolution in intervention strategies and public policy recommendations, strongly indicating that childhood obesity is a major public health problem. We excluded all records that did not meet the above criteria.

### 2.3. Study Selection

Once the inclusion/exclusion criteria were applied to each study, data on the study source, including authors and year of publication, study design, patient with obesity/overweight, family-based intervention protocol performed on patients, sample size, participant characteristics (age, height, weight, fat percentage, BMI, and gender), and final outcomes of the interventions, were mined independently by two authors (D.F.-L and S.A.) using a spreadsheet (Excel 2021 (18.0), Microsoft Inc., Seattle, WA, USA). Subsequently, disagreements were resolved through discussion until a consensus was reached or there was third-party adjudication (E.G.).

### 2.4. Quality Assessment

Methodological quality was assessed following qualitative studies via a McMaster Critical Review Form [21] independently by two authors (D.F.-L and S.A.), with disagreements being resolved by third-party evaluation (A.M.C.S.M). There were 16 evaluated items (purpose, literature review, study design, blinding, sample description, sample size, ethics and consent, validity of outcomes, reliability of outcomes, intervention description, statistical significance, statistical analysis, clinical importance, conclusions, clinical implications, and study limitations), which were rated as “1” if the criteria were fully met, “0” if they were not completely met, or “NR” in cases where information was not reported. Study scores were as follows: ≤8 points indicated poor quality; 9–10 points acceptable quality; 11–12 points good quality; 13–14 points very good quality; and ≥15 points excellent quality.

Moreover, the risk of bias in the included studies was assessed using the Cochrane risk-of-bias (RoB) [23]. This tool consists of 8 domains: random sequence generation (selection bias), allocation concealment (selection bias), blinding (performance bias and detection bias) participant, blinding (performance bias and detection bias) personnel, blinding (performance bias and detection bias) outcome assessor, incomplete outcome (attrition bias), and selective reporting (reporting bias). Two independent authors assessed the RoB, and a third author was consulted in case of disagreement.

### 2.5. Data Extraction

Once the inclusion and exclusion criteria were applied, relevant information was gathered from the chosen studies. The extracted data included the name of the primary author, publication year, country of origin, study design, sample size, participant characteristics (such as gender, age, level of physical activity, and health status), details of the intervention (duration; modality of the intervention: face-to-face or online; and information provided to parents and children), variables analyzed, and the corresponding results.

## 3. Results

### 3.1. Study Selection

**Figure 1:**
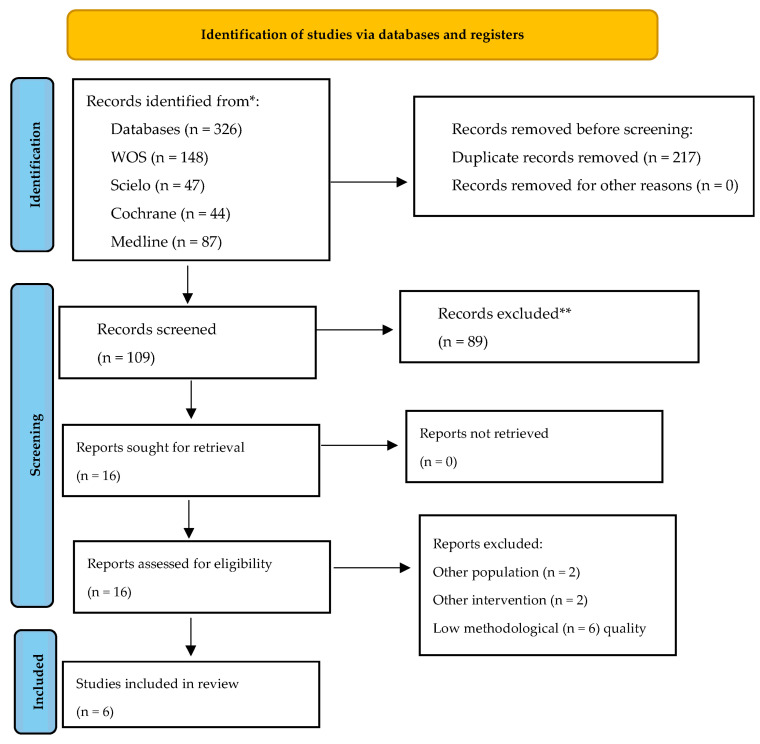
Flow diagram depicting the identification and selection processes of relevant studies according to PRISMA guidelines. * Consider, if feasible to do so, reporting the number of records identified from each database or register searched (rather than the total number across all databases/registers). ** If automation tools were used, indicate how many records were excluded by a human and how many were excluded by automation tools.

A total of 326 studies were identified. Among them, studies were initially obtained from WOS, Cochrane, Medline, and Scielo. After the exclusion of 217 duplicates, a total of 109 articles identified in databases and registries were examined. After an evaluation of the title and abstract, 16 articles were considered as potential registries. After a review of the full text and an evaluation of potential records from databases and registries as well as other sources, six studies [2,24,25,26,27,28] were included in the systematic review (Figure 1).

### 3.2. Quality Assessment

**Figure 2:**
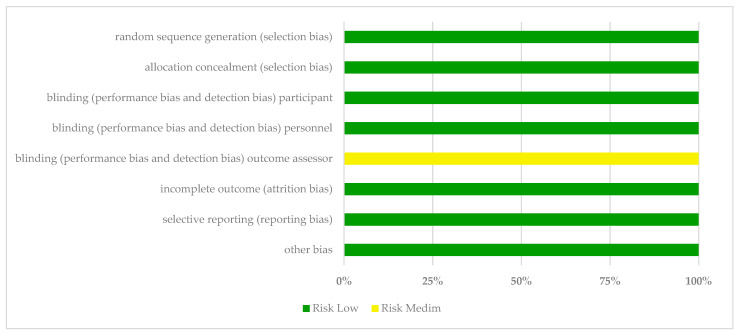
The most common problems found in the risk of bias in selected studies.

One study was considered to be of “excellent quality”, four of “very good quality”, and one of “good quality” (Table 1). Table 2 shows the results of the Cochrane risk-of-bias assessment tool applied to the studies in this review. Figure 2 presents a summary of the review authors’ judgments on each RoB item for each included study. Regarding selection bias, the allocation concealment process, blinding of participants, and blinding personnel, all studies reported an adequate method to generate a randomization sequence of participants and were judged as low RoB. Regarding the blinding of outcome assessors, six studies were classified as high RoB. For the final three items (attrition bias, reporting bias, and other bias), all studies were evaluated with low RoB.

### 3.3. Outcome Evaluation

Table 3 summarizes the contents of the studies contained in this systematic review.

### 3.4. Characteristics of the Participants and Interventions

The total size of the sample at the beginning of the intervention in this review was 808 (466 girls and 342 boys), but at the end of each of the respective protocols, the sample remained at 716, which represents an abandonment of 9.37%. Causes and *n* of particular withdrawals were not specified in some studies. Indicated causes and *n* of withdrawals are as follows: conflict with the schedule of the sessions, (*n* = 12) [26], (*n* not indicated) [27], and (*n* = 2) [28]; health problems, (*n* = 2) [24] and (*n* = 3) [26]; vacations, (*n* = 24) [24], (*n* = 1) [26], and (*n* = 1) [28]; lack of interest, (*n* = 6) [26] and (*n* = 1) [28]; unable to contact, (*n* = 6) [26]; clinic too far, (*n* = 1) [28]; family situation, (*n* not indicated) [27] and (*n* = 1) [26]; and consent withdrawal, (*n* = 4) [24]. No medications or adverse effects were reported.

The characteristics of the sample can be divided into two groups according to the classification proposed by the National Institute of Child Health and Human Development [29] in early childhood (2–5 years) and middle childhood (6–11 years). In this review, there is one study with children in the first group [26], four with a sample of the second age group [2,24,25,28], and one with a sample between the two age ranges (4–6 years) [27]. Also, the cut-off point for determining the BMI z-score category differs between the samples according to different data references for this parameter: the National Center for Health Statistics [26], the World Health Organization [28], and the International Obesity Task Force (IOTF) [30]. Table 4 shows the BMI classification criteria per study.

The intervention groups were divided into two [2] or in three [24,25,26,27,28]. The main difference with the studies that divided the sample into three was the intensity of the sessions, i.e., one of the intervention groups had a greater number of theoretical sessions or telephone support with a health professional. Only one of the studies referred to the modality of the intervention: group, individual, or mixed. Table 3 presents the characteristics of the interventions.

Regarding the duration of the intervention, studies show a wide range from 4 weeks [2] at least to 12 months [24,25,27,28]. Others also divide the protocol into stages, such as 3 months of intensive intervention and 3 months of maintenance [26]. Similarly, in the study by Wilfley et al. [25], the first 4 months correspond to behavioral weight loss treatment, and the second stage extends from the following 4 months to 12 months.

### 3.5. Intervention Effects on Anthropometry and Body Composition

The only variable analyzed by all studies was the BMI z-score [2,24,25,26,27,28]. In five studies [2,25,26,27,28], the BMI z-score was reduced in the intervention group, while in Robertson et al. [24], there was no difference in the z-score after 12 months of comparing groups. Despite this, in the intragroup comparison, the control had significantly reduced BMI z-score values compared to the intervention group.

The reduction in the BMI z-score was also assessed in studies that had more than two intervention groups, i.e., those that had more face-to-face sessions and/or a greater possibility of consulting with professionals. These particular studies registered decreases in the BMI z-score. In Wilfley et al. [25], the FBI-High group had a significantly higher decrease, with a score of 3.37, compared to FBI-Low, with a score of 6.71. The difference between the High and Low groups was the number of sessions, at 32 and 15 per week, respectively, but the content was the same.

Other studies added waist circumference [2,24,27] and anthropometric data [24,25,26,27,28], including weight, height, and height-for-age z-scores (HAZ), as secondary outcomes. They also added body fat percentage [2,24], and only one of the studies performed a blood test [28]. Regarding the waist circumference percentile, in two studies, the control group had a higher increase along the protocol compared to the intervention groups [2,27], and in Robertson et al. [24], no significant differences were observed between groups.

Body fat percentage was analyzed in three studies [2,24,28], with different results. In one of them [2], the percentage of changes was higher in the overweight intervention group, with a similar result in the study by Cohen et al. [28]. In this latter study, one of the intervention groups called ModTx displayed a significant decrease at 6 and 12 months [28]. In Robertson et al. [24], there were no differences between the groups. Regarding the body weight variable, the LAUNCH [26] or HIGH [25] groups were the ones that reported a lower body weight gain over 6 months and a higher proportion of children in percentage overweight from baseline to 12 months, respectively, compared to the control group of each study.

### 3.6. Intervention Effects on Diet and Physical Activity

In terms of lifestyle, such as nutrition and physical activity, a single study [28] provided detailed information, including the type of questionnaire used to collect details of children’s food intake and physical activity. Dietary intake was estimated using three-day food diaries (3DFDs) and the Physical Activity Questionnaire for Children (PAQ-C) [31]. The study by Robertson et al. [24] also describes the questionnaires used, in this case, the Family Eating and Activity Habits Questionnaire [32] and the Warwick-Edinburgh Mental Well-Being Scale (WEMWBS) [33].

The rest of them [2,25,26,27] were limited to providing generic recommendations when detailing the brochures and theoretical sessions provided to parents and/or children. The information available in the theoretical material given to parents and explained in the face-to-face sessions highlighted the importance of reducing the consumption of sugary drinks, avoiding snacks, and consuming five servings of fruits and vegetables. Regarding physical activity, some recommended 30 min [2] or ≥1 h of moderate to vigorous physical activity per day [26,28].

A single study [28] provided details on calories and macronutrients consumed at baseline and at 6 and 12 months. The ModTx intervention group consumed ∼340 kcal/day less compared to control. Regarding proteins, the ModTx and StnTx groups ingested less than the control. The amount of 3DFDs available for analysis decreased at 6 (only 41%) and 12 (only 33%) months. There were no differences in macronutrient amounts in the groups throughout the protocol.

## 4. Discussion

The purpose of this systematic review was to critically evaluate the effects of FBIs on children with obesity and overweight. A total of six studies met the inclusion/exclusion criteria. Intervention programs showed positive effects on reducing BMI z-scores.

### 4.1. Characteristics of the Participants and Interventions

The division of the sample according to age did not show a difference in the results of the variables analyzed. The sample in the study by Stark et al. [26] reported positive results of decreasing the BMI z-score in the same way as the studies with samples of children aged 7 to 11 years [2,25,28].

One common aspect of the interventions in this review was that they were oriented toward the motivation of children’s behaviors [25,26,27]. In one study [25], the focus of the intervention was cognitive–behavioral, where it sought to develop skills aimed at self-regulation and the prevention of relapse. In others, a socio-ecological approach was proposed [25,26,27], emphasizing the support of the family and the child’s social circle. Ek et al. [27] included the Parent Management Training—Oregon model (PMTO) technique, which aims to train parents in skills that allow them to manage children’s behaviors. The goal of PMTO is for parents to become agents of change and positively influence their children’s behavior [34]. This technique involves aspects used in other studies, such as positive reinforcement, monitoring of children’s activities, and the promotion of open communication [25,26].

The study by Ahmad et al. [2] looked at the impact of social media during the intervention. The use of social media was shown to have a positive effect, with better results than the sessions carried out face-to-face. This is due to the ease and greater availability offered by WhatsApp or Facebook compared to the face-to-face modality. In the face-to-face modality, the inconvenience and longer time involved in commuting led to a higher number of missed sessions. In another study [28], the SMART technique, an acronym for specific, measurable, achievable, relevant, and time-bound, was used to establish the personalized objectives.

Therefore, family involvement strategies, such as activities and workshops, are more effective in treating childhood obesity today because they provide consistent support, promote healthy behaviors at home, and foster a collaborative environment that reinforces positive changes [35].

### 4.2. Intervention Effects on Anthropometry and Body Composition

The cut-off points for determining obesity or overweight and the reference used to set those points make it difficult to compare the studies. Only two studies used the 85th percentile to diagnose overweight, and one of them [28] used the WHO criteria, while the others did not provide this information [25].

The difference between the intervention groups is explained not only by the duration and number of weekly sessions but also by the follow-up and goal-setting [25]. The intervention groups that received the most assistance, either with a greater number of visits or weekly sessions, were the ones with the greatest reductions in the BMI z-score throughout the protocol [25,26,27,28]. Sessions with specialized content, with well-targeted goals in weight loss and increased feedback from health professionals, contributed to a positive effect on health [25].

The other variables analyzed in the studies focused on fat mass percentage and waist circumference. For both variables, the results were different. For waist circumference, two of the three studies [2,27] recorded decreases in values in the intervention groups, while the third study [24] showed no differences between groups. The study by Robertson et al. [24] lasted 10 weeks, while the two that recorded reductions in the percentage of fat mass presented durations of 4 weeks [2] and 12 months [27]. With the data from these three studies with different durations and without detailed specifications of nutritional or physical activity recommendations, it is not possible to conclude whether the duration of the intervention plays a relevant role in the process, as well as the technical guidelines addressed by health professionals. One study [2] highlighted the importance of the support and motivation that social networks provide in modifying healthy habits.

The same situation occurred for the values of % fat mass in the three studies. One did not register differences between the groups [24], while the other two [2,28] reported decreases in the intervention groups. Similarly, the discrepancy in the results does not allow us to determine the relevance of the duration of the intervention or to determine the influence of nutritional material provided by health professionals.

Altogether, the BMI z-score is generally the best technique for determining childhood obesity because it adjusts BMI for age and sex, allowing for comparisons across different ages and growth patterns. It is widely used in clinical and research settings, providing a standardized measure to assess obesity in children [30].

### 4.3. Intervention Effects on Diet and Physical Activity

Intervention studies looked at the impact of parents’ involvement in programs regarding eating habits [25,26] or behavior and support of the children during the protocol [24]. The nutritional recommendations focused on the interpretation of food labeling [24,25], snack time [2,24,26], limiting portion size [26], shopping in the markets [24,25], eating foods low in saturated fat [28], the intake of five servings of fruits and vegetables [2,26], and reducing the intake of sugar-sweetened beverages [2,26]. Other studies established more general recommendations, such as encouraging healthy behaviors without providing details on how to perform them [25,27]. A single study [28] looked at calories, macronutrient distribution, and portion details of fruits, vegetables, meat, dairy, and cereals. In this study, the two intervention groups (StnTx and ModTx) maintained lower energy consumption compared to the control at the beginning of the protocol. In the same way, these two intervention groups were closer to the nutritional guidelines used in the protocol at the end of the study.

The absence of details on the specific nutritional recommendations used makes it difficult to establish the true extent of this factor in decreasing BMI z-scores. In addition, in some of them, it was clarified that the diet was personalized, so it is not possible to analyze the intake knowing only the nutritional guide used for the design of the intervention. Similarly, the difficulty of compiling the nutritional questionnaires used throughout the protocol [28] does not allow an adequate comparison to be made of the quantities of food and the quality of the diet consumed during the intervention.

The same is true for physical activity and lifestyle. In one study, the proposal is to perform at least 30 min of moderate to vigorous activity [2], while in another, the duration is at least 1 h of moderate to intense physical exercise [26,28]. Regarding lifestyle, information is related to reducing screen time to less than two hours [2,26] and the presence of television in children’s bedrooms [26]. The remaining studies [24,25,27] mention physical activity as part of the intervention programs but do not provide information on the duration, frequency, or intensity.

Therefore, future interventions aiming to explain the decrease in BMI z-scores in childhood obesity need to collect dietary data including total caloric intake (kcal) and the number of servings of different food groups. These data help to assess the overall energy balance and nutritional quality of the diet, which are crucial factors in weight management and BMI z-score reduction. At the same, these data will provide information for planning more efficient and optimal physical activity programs [36]. In addition, due to limitations of the BMI z-score, it is recommended for future research to record additional parameters, such as waist circumference, fat mass, and fat-free mass, according to [37,38,39].

## 5. Limitations and Strengths

Further research is necessary to establish a conclusive understanding of specific aspects of nutrition, such as energy demand and macronutrient distribution, as well as recommendations for physical activity in duration, intensity, and frequency in children who are obese or overweight. The lack of common guidelines in interventions makes it difficult to compare between studies. These studies differ in duration, intervention modality, number of sessions, nutritional guidelines, and indications for physical activity. Similarly, in some cases, the details of the protocols are not provided, making it difficult to establish the relationships between the decrease in the values obtained and the factors that affect this result. Another drawback to the analysis was the criteria used to classify a child as obese or overweight. Some provided the information, while others used standards from organizations or institutions with different classification categories. Finally, an instrumental limitation is the low number of studies. This limitation was because we only included original studies that were randomized controlled clinical trials with high methodological quality (≤8 on the McMaster Critical Review Form) and that evaluated physical and/or anthropometric changes as primary or secondary outcomes in the 10 last years.

## 6. Practical Applications

Different national and international reports and surveys warn of the increase in overweight and obesity in children and adolescents around the world, which poses a risk to their physical and mental health. In this sense, controlling obesity and overweight in childhood is essential to protect their health, improve their quality of life, prevent chronic diseases, and reduce the social and health costs that they entail for countries’ economies. The established recommendations are based on family-based multicomponent behavioral treatment programs that have proven to be the most effective [2,24,25,26,27,28]. However, to ensure their effectiveness, we indicate several aspects that can be implemented in the selected protocols [2,24,25,26,27,28] or can be considered in future research. First, FBIs must begin as early as possible, and the intervention should not be limited only to the child or adolescent. Parents should also be involved as well as other family members. However, the level of participation of family members will depend on the child’s stage of development. The choice of the final program depends on the criteria of the health professional. Second, it is necessary to implement a program that can better adjust to the characteristics of each child and the resources of each family. Third, it is advisable to consider gender, age, and family culture before opting for one program or another.

Involving family members in the treatment of children with obesity or overweight is crucial because it creates a supportive environment that promotes healthy habits and lifestyle changes. Family members can serve as role models, provide encouragement, and help establish routines that prioritize nutritious meals and physical activity. By working together, families can address underlying issues, such as unhealthy eating patterns or sedentary behaviors, and implement sustainable changes that benefit the child’s overall well-being.

## 7. Conclusions

Family-based interventions reported improvements in BMI z-scores and, in some cases, in waist circumference and percentage total fat mass. This type of intervention focuses on changing the behavior of children with obesity or overweight, as well as parental support for the acquisition of healthy behaviors. Regarding nutritional and physical activity guidelines, general recommendations can be considered: the inclusion of fruits and vegetables, reducing sugary drinks, reducing screen time, and engaging in at least 30 min to 1 h of physical activity. Based on the results, this systematic review indicates that more evidence is needed to give clear recommendations on dietary and physical activity aspects in cases of childhood obesity or overweight.

## Figures and Tables

**Table 1:** Results of the methodological quality assessment of included studies—McMaster Critical Review Form for Quantitative Studies [21].

Study	Items																Total	%	Quality Score
	1	2	3	4	5	6	7	8	9	10	11	12	13	14	15	16			
Ahmad et al. [2], 2018	1	1	1	1	1	1	0	1	1	1	1	1	0	1	1	0	15	93.75	E
Cohen et al. [28], 2016	1	1	1	1	1	1	0	1	1	0	0	1	1	1	1	1	13	81.25	VG
Ek et al. [27], 2019	1	1	1	1	1	1	0	1	1	0	0	1	1	1	1	1	13	81.25	VG
Robertson et al. [24], 2017	1	1	1	1	1	1	0	1	1	0	0	1	1	1	1	1	13	81.25	VG
Stark et al. [26], 2018	1	1	1	1	1	1	0	0	1	0	0	1	1	1	1	1	12	75	G
Wilfley et al. [25], 2017	1	1	1	1	1	1	0	1	1	0	0	1	1	1	1	1	13	81.25	VG

Abbreviations: 0 = not fulfilled criterion; 1 = fulfilled criterion; E = excellent; VG = very good.; G = good; item 1: study purpose; item 2: literature review; item 3: study design; item 4: blinding; item 5: sample description; item 6: sample size; item 7: ethics and consent; item 8: validity of outcomes; item 9: reliability of outcomes; item 10: intervention description; item 11: statistical significance; item 12: statistical analysis; item 13: clinical importance; item 14: conclusions; item 15: clinical implications; and item 16: study limitations.

**Table 2:** Cochrane risk-of-bias assessment [23].

	Random Sequence Generation (Selection Bias)	Allocation Concealment (Selection Bias)	Blinding (Performance Bias and Detection Bias) Participant	Blinding (Performance Bias and Detection Bias) Personnel	Blinding (Performance Bias and Detection Bias) Outcome Assessor	Incomplete Outcome (Attrition Bias)	Selective Reporting (Reporting Bias)	Other Bias
Ahmad et al. [2], 2018	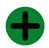	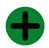	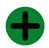	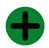	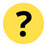	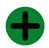	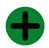	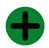
Cohen et al. [28], 2016	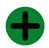	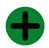	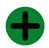	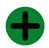	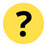	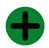	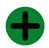	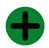
Ek et al. [27], 2019	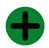	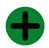	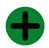	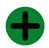	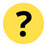	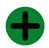	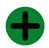	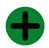
Robertson et al. [24], 2017	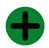	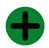	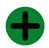	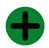	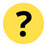	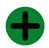	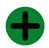	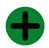
Stark et al. [26], 2018	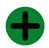	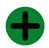	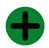	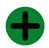	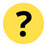	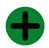	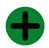	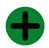
Wilfley et al. [25], 2017	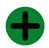	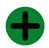	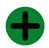	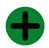	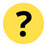	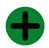	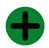	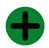

Note: Risk of bias summary: based on authors’ judgments about each risk of bias item for each included study. (+) = low risk of bias; (?) = unclear risk of bias.

**Table 3:** Studies included in the systematic review of family-based treatment for childhood obesity.

First Author, Year of Publication, and Country	Study Design	Participants: Group Name, Number (Gender), Anthropometric Characteristics, and Withdrawals	Variable	Outcomes	Conclusions
Ahmad et al. [2], 2018 Malaysia	Randomized clinical trial	REDUCE: *n* = 67 (39 girls and 28 boys) Age (mean ± SD): 9.6 ± 1.2 years Height (mean ± SD): 136.1 ± 8.6 cm Weight (mean ± SD): 47.0 ± 10.5 kg BMIz (mean ± SD): 2.05 ± 0.40 BMI (mean ± SD): 25.2 ± 3.5 % BF (mean ± SD): 37.87 ± 4.20 Waist circumference (mean ± SD): 90.21 ± 7.98th CG: *n* = 67 (37 girls and 30 boys) Age (mean ± SD): 9.6 ± 1.2 years Height (mean ± SD): 135.6 ± 9.2 cm Weight (mean ± SD): 48.2 ± 12.0 kg BMIz (mean ± SD): 2.11 ± 0.4 BMI (mean ± SD): 25.7 ± 3.9 % BF (mean ± SD): 37.63 ± 4.09 Waist circumference (mean ± SD): 91.28 ± 7.04th Study withdrawals: 0	BMI z-score % total body fat Waist circumference percentile	REDUCE vs. CG ↓* BMIz REDUCE vs. CG ↓* % total body fat REDUCE vs. CG ↓* Waist circumference percentile	The 4-month REDUCE intervention program was effective in reducing infant adiposity.
Cohen et al. [28], 2016 Canada	Randomized clinical trial	StnTx: *n* = 25 (14 girls and 11 boys) Age (mean ± SD): 7.7 ± 0.8 years BMI (mean ± SD): 25.2 ± 8.4 %BF (mean ± SD): 38.5 ± 5.5 Waist circumference (cm) (mean ± SD): 81.3 ± 8.4 Study withdrawals: 2 ModTx: *n* = 25 (15 girls and 10 boys) Age (mean ± SD): 8.1 ± 0.7 years BMI (mean ± SD): 24.1 ± 2.8 %BF (mean ± SD): 36.8 ± 5.5 Waist circumference (cm) (mean ± SD): 81.6 ± 8.7 Study withdrawals: 1 Control: *n* = 28 (16 girls and 12 boys) Age (mean ± SD): 7.7 ± 0.8 years BMI (mean ± SD): 24.1 ± 3.2 %BF (mean ± SD):36.2 ± 15.5 Waist circumference (cm) (mean ± SD): 80.7 ± 9.1 Study withdrawals: 2	∆BAZ ∆%BF ∆FM LM Waist circumference Macronutrient intake Saturated fatty acids	ModTx vs. CG ↓* ∆BAZ ↓* ∆% BF ↓* ∆FM CG vs. ModTx ↑* ∆Waist circumference ↑* ∆FM of trunk ↑* LM in all groups FMI ModTx ≈ CG Macronutrient intake ≈ in all groups Saturated fatty acids ≈ in all groups (at 3 months)	The intervention based on the Canadian guidelines on diet and PA had effects on reducing adiposity in children with O/SP
Ek et al. [27], 2019 Sweden	Randomized clinical trial	Booster: *n* = 44 (19 girls and 25 boys) Age (mean ± SD): 5.2 ± 0.8 years BMIz (mean ± SD): 3.0 ± 0.5 BMI (mean ± SD): 21.4 ± 1.5 Waist circumference (cm) (mean ± SD): 65.1 ± 4.3 Study withdrawals: 18 No Booster: *n* = 43 (23 girls and 20 boys) Age (mean ± SD): 5.2 ± 0–9 years BMIz (mean ± SD): 3.1 ± 0.7 BMI (mean ± SD): 21.9 ± 2.3 Waist circumference (cm) (mean ± SD): 67.6 ± 6.5 Study withdrawals: 13 CG: *n* = 87 (42 girls and 45 boys) Age (mean ± SD): 5.3 ± 0.7 years BMIz (mean ± SD): 2.9 ± 0.6 BMI (mean ± SD): 21.3 ± 1.7 Waist circumference (cm) (mean ± SD): 66.9 ± 6.2 Study withdrawals: 16	BMIz Waist circumference	Booster + No booster vs. CG ↓ BMIz CG vs. Booster + No booster ↑* Waist circumference	A parent-only treatment with boosters exceeded standard care for the treatment of obesity in preschoolers.
Robertson et al. [24], 2017 United Kingdom	Randomized clinical trial	FFH: *n* = 56 (32 girls and 24 boys) Age (mean ± SD): 9.44 ± 1.47 years BMIz (mean ± SD): 2.7 ± 0.63 BMI (mean ± SD): 25.92 ± 4.57 Study withdrawals: 12 CG: *n* = 59 (27 girls and 32 boys) Age (mean ± SD): 9.25 ± 1.61 years BMIz (mean ± SD): 2.68 ± 0.69 BMI (mean ± SD): 25.08 ± 3.57 Study withdrawals: 20	BMIz Waist circumference	↓ BMIz in all groups CG: ↓* BMIz, FFH: ↓ BMIz FFH ≈ CG waist circumference	FFH was neither effective nor cost-effective for the treatment of obesity compared to usual care.
Stark et al. [26], 2018 United States	Randomized clinical trial	LAUNCH: *n* = 57 (25 girls and 32 boys) Age (mean ± SD): 55.10 ± 12.07 months BMIz (mean ± SD): 2.41 ± 0.53 Height (mean ± SD): 111.02 ± 8.71 cm Weight (mean ± SD): 26.15 ± 6.16 kg Study withdrawals: 14 MI: *n* = 56 (39 girls and 17 boys) Age (mean ± SD): 55.0 ± 10.67 months BMIz (mean ± SD): 2.41 ± 0.56 Height (mean ± SD): 111.62 ± 8.04 cm Weight (mean ± SD): 25.91 ± 5.02 kg Study withdrawals: 10 CG: *n* = 54 (32 girls and 22 boys) Age (mean ± SD): 55.30 ± 11.06 months BMIz (mean ± SD): 2.48 ± 0.70 Height (mean ± SD): 110.70 ± 7.92 cm Weight (mean ± SD): 25.97 ± 5.47 kg Study withdrawals: 4	BMIz	LAUNCH vs. CG ↓* BMIz ↓ BMI in CG for 6 months.	A 6-month intensive (LAUNCH) behavioral skills-based intervention (LAUNCH) was needed to reduce obesity in preschool children.
Wilfley et al. [25], 2017 United States	Randomized clinical trial	SFM + H: *n* = 59 (37 girls and 22 boys) Age (mean ± SD): 9.5 ± 1.3 years Study withdrawals: 4 SFM + L: *n* = 56 (36 girls and 20 boys) Age (mean ± SD): 9.4 ± 1.2 years Study withdrawals: 2 Control: *n* = 57 (33 girls and 24 boys) Age (mean ± SD): 9.5 ± 1.3 years Study withdrawals: 6	BMIz % Overweight	SFM + H vs. CG ↓* BMIz SFM + H vs. CG ↓* % Overweight SFM + H vs. CG ↑* Proportion of children who decreased ≥9 units in the % of overweight	The content of the SFM + H specialized intervention improved weight outcomes in children.

Symbols and abbreviations used: ↑: increase; ↓: decrease; *: statistically significant; ≈: equal; ≥: greater than or equal to; ±: more/less; ∆: increments; vs.: with respect to; BAZ: BMIz for age; %BF: body fat percentage; BMI: body mass index; BMIz: BMI z-score; CG: control group; FFH: families for health; FM: fat mass; FMI: fat mass index; LM: lean mass; MI: motivational interviewing; ModTx: modified intervention; *n*: sample size; SFM + H: social facilitation maintenance high; SFM + L: social facilitation maintenance low; StnTx: standard intervention; and th: percentile.

**Table 4:** Classification of the sample according to BMI z-score category.

Reference	BMI z-Score Category	Value Range	International Organization/Institution (for BMI Reference)
Ahmad et al. [2], 2018	Overweight	N/D	N/D
	With BMI z-score of more than 1 standard deviation		
Cohen et al. [28], 2016	Overweight	85–97 percentile	World Health Organization
	Obese	>97 percentile	
Ek et al. [27], 2019	With obesity	N/D	International Obesity Task Force (IOTF)
Robertson et al. [24], 2017	Overweight	≥91st percentile BMI	N/D
	Obese	≥98th percentile BMI	
Stark et al. [26], 2018	Overweight	BMI percentile for age and sex ≥95	National Center for Health Statistics
	Obese	No more than 100% above the median BMI	
Wilfley et al. [25], 2017	Overweight	≥85th percentile	N/D
	Obese	BMI ≥ 25	

Abbreviations: N/D: no data; BMI: body mass index.

## Data Availability

Not applicable.

## Appendix A. Search Strategy

**Database**	**Keywords**	**Records Identified**
PubMed	((childhood obesity [Title/Abstract] OR Obesity [Title/Abstract] OR children [Title/Abstract]) AND (‘physical activity [Title/Abstract] OR ‘exercise’ [Title/Abstract]) AND (Diet [Title/Abstract] OR exercise [Title/Abstract]) Filters: Randomized Controlled Trial, from 2014–2024, in the last 10 years.	87
Scielo	(“childhood obesity” [Title/Abstract] OR “obesity” [Title/Abstract] OR “children” [Title/Abstract]) AND (“physical activity” [Title/Abstract] OR “exercise” [Title/Abstract]) AND (“intervention” [Title/Abstract] OR “family-based intervention” [Title/Abstract]). In Title Abstract Keyword in All Text—Publication Year from 2014 to 2024. Filters: Full text, Trial, in the last 10 years.	44
Web of Science	Childhood obesity OR obesity OR children (Topic) and physical activity OR exercise (Topic) and intervention OR family-based intervention (Topic) and 2024 or 2023 or 2022 or 2021 or 2020 or 2019 or 2018 or 2017 or 2016 or 2015 or 2014 (Publication Years) and Article (Document Types)	148
Cochrane	(Childhood obesity OR obesity OR children): ti,ab,kw AND (physical activity OR exercise): ti,ab,kw AND (intervention OR family-based intervention): ti,ab,kw’ with Cochrane Library publication date Between Jan 2014 and June 2024, in Trials (word variations have been searched)	47
